# Immune complex-induced apoptosis and concurrent immune complex clearance are anti-inflammatory neutrophil functions

**DOI:** 10.1038/s41419-021-03528-8

**Published:** 2021-03-19

**Authors:** Utsa Karmakar, Julia Y. Chu, Kruthika Sundaram, Anne L. Astier, Hannah Garside, Carsten G. Hansen, Ian Dransfield, Sonja Vermeren

**Affiliations:** 1 The Centre for Inflammation Research, Institute for Regeneration and Repair, The University of Edinburgh, Edinburgh, UK; 2 Centre for Physiopathology Toulouse-Purpan, INSERM U1043, CNRS U5282, Toulouse University, Toulouse, France

**Keywords:** Apoptosis, Endocytosis, Cell death and immune response, Neutrophils

## Abstract

Persistent neutrophilic inflammation drives host damage in autoimmune diseases that are characterized by abundant immune complexes. Insoluble immune complexes (iICs) potently activate pro-inflammatory neutrophil effector functions. We and others have shown that iICs also promote resolution of inflammation via stimulation of neutrophil apoptosis. We demonstrate here that iICs trigger FcγRIIa-dependent neutrophil macropinocytosis, leading to the rapid uptake, and subsequent degradation of iICs. We provide evidence that concurrent iIC-induced neutrophil apoptosis is distinct from phagocytosis-induced cell death. First, uptake of iICs occurs by FcγRII-stimulated macropinocytosis, rather than phagocytosis. Second, production of reactive oxygen species, but not iIC-internalization is a pre-requisite for iIC-induced neutrophil apoptosis. Our findings identify a previously unknown mechanism by which neutrophils can remove pro-inflammatory iICs from the circulation. Together iIC clearance and iIC-induced neutrophil apoptosis may act to prevent the potential escalation of neutrophilic inflammation in response to iICs.

## Introduction

Neutrophils, the most abundant circulating leukocytes are vital for host immunity, phagocytosing and killing bacteria and fungi^1,2^. The short-lived neutrophil originates in the bone marrow and is subsequently released into the circulation. Unless recruited to peripheral sites in response to specific stimuli, neutrophils circulate in the bloodstream for ~1 day before homing back to the bone marrow where they undergo apoptosis prior to clearance by resident macrophages^3,4^. Activating signals trigger neutrophil transition to a reversible, primed state which precedes full activation. Priming triggers heightened responsiveness to certain stimuli and physiological changes including delayed apoptosis^5^. Neutrophil apoptosis can be induced by diverse triggers; for example, having phagocytosed and killed microbes, neutrophils undergo phagocytosis-induced cell death [PICD^6–8^]. Apoptotic neutrophils generate ‘find-me’ and ‘eat-me’ signals that direct their efferocytosis by phagocytic macrophages and generates pro-resolution signals^4^.

Neutrophils are key players of the inflammatory response, which is subject to tight control mechanisms. When these go awry, neutrophils are activated indiscriminately. Autoimmune diseases including rheumatoid arthritis are characterized by abundant autoantibodies that form immune complexes (ICs). IC-driven Fc receptor activation promotes neutrophil activation, contributing to host tissue damage^9–11^. Depending on antibody subclass and antigen–antibody ratio, soluble or insoluble immune complexes (iICs) form, which circulate in the bloodstream and are present in other bodily fluids, e.g., synovial fluid. Complement deposition on small soluble ICs enables efficient binding to erythrocyte complement receptor 1, which promotes efficient clearance of circulating soluble ICs by resident macrophages in the liver and spleen^12–15^. Larger ICs, which are cleared less efficiently, also circulate and are more likely to deposit in tissues and activate Fc receptor-expressing immune cells, e.g., neutrophils, enhancing inflammation and disease progression. Neutrophils constitutively express the low affinity receptors for IgG FcγRII (CD32) and FcγRIII (CD16); they also inducibly express the high affinity receptor FcγRI (CD64), which is expressed at very low levels on unprimed neutrophils^16^. Ligation of neutrophil FcγRs potently triggers pro-inflammatory functions, including the generation of reactive oxygen species (ROS), degranulation and cytokine production. Whereas soluble ICs stimulate only primed neutrophils, iICs also activate unprimed neutrophils^17^. We and others have previously shown that in addition to activating these pro-inflammatory neutrophil functions, iICs also promote neutrophil apoptosis^18,19^.

We demonstrate here how iIC-induced neutrophil apoptosis coincides with neutrophil-mediated clearance of iICs. iIC-induced neutrophil apoptosis is distinct from PICD, and controlled by a different mechanism. Neutrophils do not phagocytose iICs, but rather internalize them by FcγRII-stimulated macropinocytosis. Our findings suggest that iIC-induced neutrophil apoptosis represents an efficient anti-inflammatory mechanism for clearance of iICs from the circulation.

## Materials and methods

### Reagents

Unless indicated otherwise, reagents of the lowest possible endotoxin level were from Sigma-Aldrich (Gillingham, UK). Tissue culture reagents were from Gibco (Thermo Fisher Scientific, Loughborough, UK) and percoll from GE Healthcare (Amersham, UK). For inhibitors and antibodies used see data supplement.

### Isolation of human peripheral blood neutrophils

Neutrophils were prepared as described^18^ to purity >95% according to Diff-Quik (Thermo Scientific) stained cytospins.

### Insoluble immune complexes

iICs were prepared from human serum albumin (HSA) and rabbit polyclonal IgG to HSA as described^18,20^. Titration experiments determined iIC concentrations required to trigger substantial responses (not shown).

### Analysis of neutrophil apoptosis

Neutrophil apoptosis and loss of plasma membrane integrity were analyzed by flow cytometry (FACSCalibur, BD Biosciences, Oxford, UK or Attune NxT, Thermo Fisher Scientific, Loughborough, UK) of FITC-annexin V (Roche, Welwyn Garden City, UK) and propidium iodide-stained neutrophils^18^. Activated caspase-3 was measured using an AF488-coupled antibody specific for cleaved human caspase-3 according to the manufacturer’s instructions. Data were analyzed using FlowJo (TreeStar; version 6.4.7) or FCS Express 7 (De Novo).

### Diisopropyl fluorophosphate (DFP) treatment of neutrophils

Neutrophils were incubated with 7 mM DFP for 5 min at room temperature in a fume hood, washed twice with PBS and used in experiments as detailed.

### Western blotting

Neutrophils were lysed with ice-cold lysis buffer [20 mM Tris-HCl (pH 7.5), 150 mM EDTA, 1 mM EGTA, 1% Triton X-100, 2.5 mM sodium pyrophosphate, 1 mM β-glycerophosphate, 1 mM sodium orthovanadate, 0.1 mM PMSF, 10 μg/ml each of antipain, aprotinin, pepstatin A and leupeptin]. The detergent-soluble material was boiled in reducing sample buffer, subjected to SDS-PAGE and wet transferred to PVDF membrane (Millipore) for detection of proteins of interest using suitable antibodies. For IgG degradation assays, membranes were incubated with a HRP-coupled anti-rabbit secondary antibody. Blots were developed using chemoluminescence (Millipore).

### Internalization assays

Neutrophils were resuspended in Dulbecco’s PBS with CaCl_2_ and MgCl_2_, supplemented with 1 g/L glucose and 4 mM sodium bicarbonate (PBS^++^), treated with inhibitor or vehicle as indicated and stimulated with iICs (sometimes fluorescently labelled) or opsonized latex beads at 37 °C. Negative controls were kept on ice. Cells were analyzed by flow cytometry (Attune Nxt; analysis by FCS Express 7), or allowed to adhere to (sometimes electrostatic) slides on ice prior to paraformaldehyde fixation and staining. For live imaging, freshly isolated neutrophils were incubated with Cell Mask Deep Red (Thermo Fisher Scientific) and allowed to attach to coverslips at 37 °C before stimulation with fluorescently labelled iICs.

### Particles

Zymosan was opsonized with autologous serum for 30 min followed by PBS washes prior to use in experiments. Latex beads were opsonized with polyclonal rabbit IgG as per the manufacturer’s instructions.

### ROS production

ROS production was measured indirectly using chemiluminescence production by 5 × 10^5^ neutrophils per well at 37 °C in luminescence-grade 96-well plates (Nunc, Thermo Fisher Scientific) in a Cytation plate reader (BioTek, Swindon, UK) as described^18,21^, with neutrophils incubated with 150 μM luminol for the analysis of internal ROS production, and with 150 μM luminol and 18.75 U/ml horseradish peroxidase for the analysis of total ROS. Data output was in light units/seconds.

### Image processing

Image deconvolution of raw confocal images obtained with the Leica SP8 confocal was performed using Huygens Professional software (Scientific Volume Imaging). Confocal stacks were routinely obtained, suitable optical sections selected and pseudo colouring applied using ImageJ (NIH). Raw movie files were analyzed with Imaris (Bitplane) software without deconvolution.

### Statistical analysis

Power calculations were not performed as part of this work. Statistical analysis was performed with Graph Pad Prism 8. Where data met the assumptions for parametric tests, one-way ANOVA or RM one-way ANOVA was performed with Dunnett’s multiple comparison test; otherwise, Kruskal–Wallis test was performed. For kinetic experiments, the area under the curve was used for analysis. Individual values are plotted throughout to show the variance of each dataset. *p* values < 0.05 were deemed statistically significant. Comparisons shown relate to the activated control condition of each graph.

## Results

### As well as inducing neutrophil apoptosis, iICs are internalized by neutrophils in a PI3K-dependent fashion

**Fig. 1:**
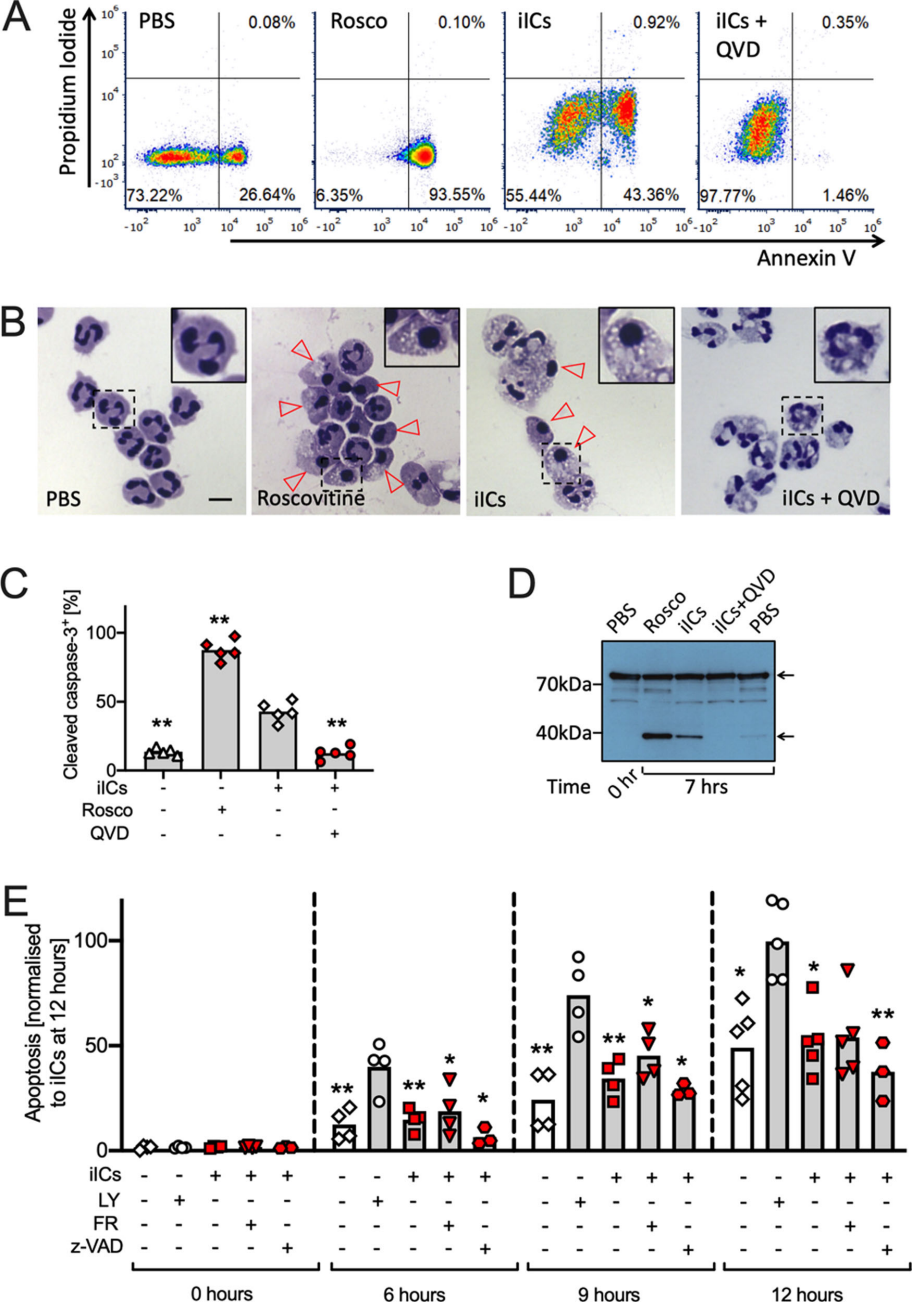
iIC stimulation induces neutrophil apoptosis. Neutrophils were pre-incubated with inhibitors or vehicle as indicated (Rosco, roscovitine, CDK inhibitor; QVD, Q-VD-OPh hydrate and z-VAD, z-VAD-FMK, pan-caspase inhibitors; LY, LY294002, pan-PI3K inhibitor; FR, FR180204, Erk inhibitor) at 37 °C for 10 min, and stimulated with 10 μg/ml iICs (HSA anti-HSA) or vehicle in IMDM supplemented with 10% autologous serum and cultured at 37 °C. After 6 h **A** cells were stained with annexin V and propidium iodide and analyzed by flow cytometry (Fig. S1A for gating). **B** Cytospins were prepared and cytoplasm and nuclei stained. Brightfield images were taken (×40 magnification; Evos imaging system). Arrowheads identify some apoptotic cells with characteristic condensed nuclei. Boxed cells are shown enlarged in inset panels. Scale bar, 10 μm. **C** Cleaved caspase-3 was detected by flow cytometry in neutrophils that had been treated as indicated and cultured for 7 h (Fig. S1D, E for gating and additional controls). **D** Cell lysates were prepared and processed for Western blotting to detect full-length gelsolin and its cleavage product (arrows). **A**, **B**, **D** Representative examples from ≥ 3 separately performed experiments are presented. **E** Graphical representation of neutrophil apoptosis (as identified in **A**) under the indicated conditions and times. **C**, **E** Each symbol represents the value obtained in a separate experiment. Raw data were subjected to analysis by one-way ANOVA and multi comparisons post-hoc test, comparing all conditions to iIC-stimulated neutrophils. *p < 0.05, **p < 0.01.

We previously reported that iICs induce neutrophil apoptosis by engaging a non-canonical PI3K signalling cascade, altering the ratio of pro- and anti-apoptotic Bcl2 family proteins towards the pro-apoptotic Bax^18^. Specifically, iIC stimulation of neutrophils, and treatment with a positive control, the cyclin-dependent kinase inhibitor roscovitine^22,23^ caused caspase-dependent plasma membrane phosphatidylserine exposure (Fig. 1A; Fig. S1A, B). Apoptotic neutrophils were characterized by chromatin condensation, resulting in loss of the characteristic multilobed neutrophil nuclear morphology (Fig. 1B; Fig. S1C). Stimulating neutrophils with iICs resulted moreover in activation of the executioner caspase-3 (Fig. 1C; Fig. S1D–F) and in gelsolin cleavage (Fig. 1D), further markers of apoptotic cell death^24,25^. The induction of apoptosis by iICs occurred over a timeframe of 6–12 h (Fig. 1E; ref. ^18^) with cells beginning to lose plasma membrane integrity at later times (Fig. S1A).

**Fig. 2:**
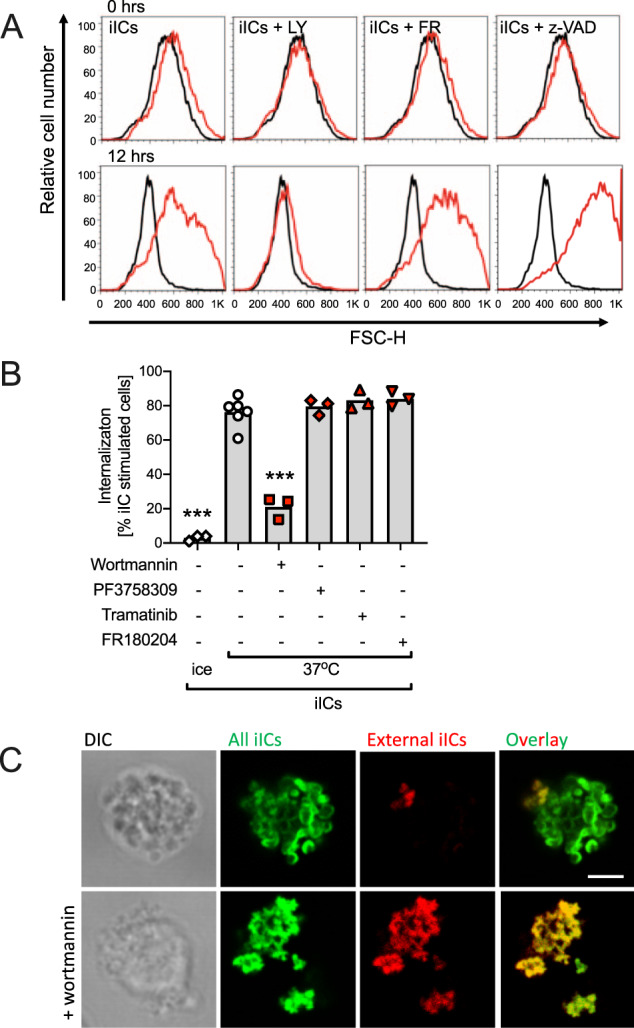
Neutrophils internalize iICs in a PI3K-dependent fashion. Neutrophils were pre-incubated with small molecule inhibitors or vehicle (LY, LY274002, wortmannin, pan-PI3K inhibitors; FR, FR180204; z-VAD, z-VAD-FMK; PF3758309, Pak inhibitor; Tramatinib, Mek inhibitor) at 37 °C for 10 min and stimulated with 10 μg/ml iICs (HSA anti-HSA) in IMDM supplemented with 10% autologous serum and cultured at 37 °C. **A** Singlets were gated and analyzed by flow cytometry at the indicated times. Representative forward scatter histograms are plotted. For ease of viewing, plots of vehicle-treated cells obtained at the indicated times (black) are shown in all iIC-stimulated conditions (red). A representative example is shown of ≥3 independent experiments performed. (**B** + **C**) Following vehicle or inhibitor treatment, neutrophils were stimulated for 30 min with (**B**) AF488-labelled iICs on ice or at 37 °C in suspension. Neutrophils were then placed on ice and attached iICs labelled with an AF-647-conjugated secondary antibody, and cells analyzed by flow cytometry. Internalized iICs are plotted. Each symbol represents the average value obtained in a separate experiment. Raw data were subjected to analysis by one-way ANOVA with multi comparisons post-hoc test. ***p < 0.001; **C** neutrophils were stimulated with unlabelled iICs at 37 °C in suspension, allowed to adhere to glass slides on ice, and fixed. External iICs were labelled using an AF568-conjugated secondary antibody. Then, after permeabilization, all iICs were labelled with an AF488-conjugated secondary antibody. Representative optical sections obtained using a Zeiss LSM780 confocal microscope with 63× objective are shown. Scale bar, 2 μm.

When analyzing neutrophil apoptosis using flow cytometry, we observed that iIC stimulation caused a reproducible shift in the forward scatter properties of neutrophils (Fig. 2A). Increased forward scatter was not observed with iIC-stimulated neutrophils in which PI3K signalling had been inhibited by LY294002, while inhibiting other components of the pathway (Fig. 2A and not shown) or a pan-caspase inhibitor did not have this effect. Since an altered forward scatter is indicative of changes in neutrophil morphology^26^, we hypothesized that neutrophils internalize iICs in a fashion that is dependent on PI3K but not Pak, Mek, Erk or caspases.

To test this possibility, we pre-incubated neutrophils with inhibitors specific for PI3K, Pak, Mek or Erk or a vehicle control prior to stimulation with fluorescently labelled iICs, and analyzed iICs internalization by flow cytometry (Fig. 2B; Fig. S2A, C, D). These experiments showed that iICs were internalized in a PI3K-, but not a Pak-, Mek- or Erk-dependent fashion. Inhibition of PI3K inhibited iIC internalization, but not iIC binding to neutrophils (Fig. 2C; Fig. S2C, D), with the concentration of the stable pan-PI3K inhibitor LY294002 used in the apoptosis assay causing significant, albeit sub-maximal inhibition of iIC internalization (Fig. S2B, C). We concluded that iIC internalization by neutrophils and iIC-dependent induction of neutrophil apoptosis are regulated by distinct pathways downstream of PI3K.

### iIC-induced neutrophil apoptosis is distinct to phagocytosis-induced cell death

**Fig. 3:**
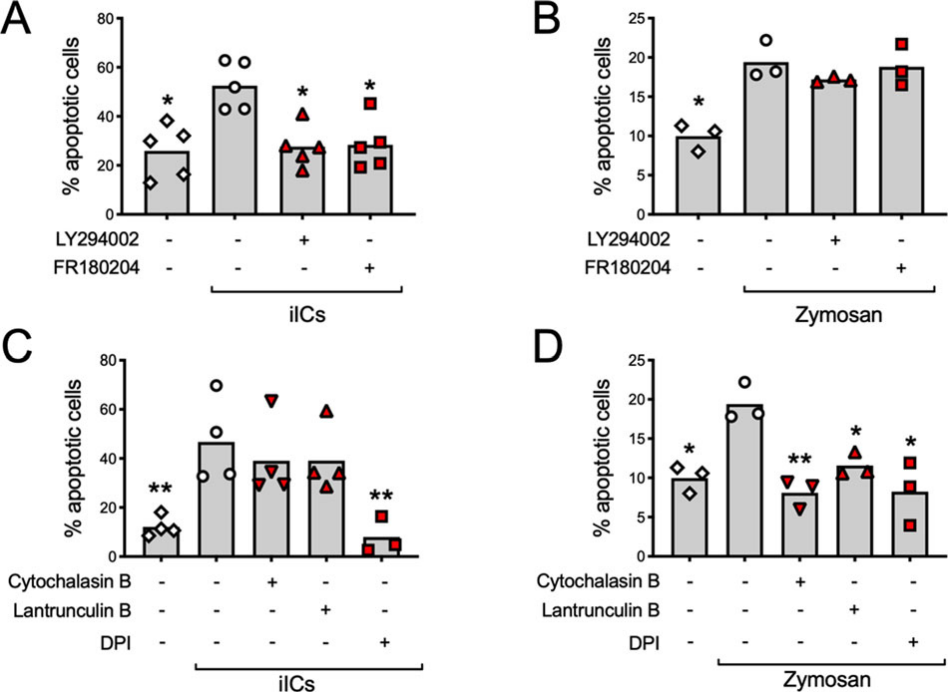
iIC-induced neutrophil apoptosis is not PICD. Neutrophils were pre-incubated with small molecule inhibitors or vehicle at 37 °C for 10 min as indicated, stimulated with (**A**, **C**) 10 μg/ml iICs (HSA anti-HSA) or (**B**, **D**) 3 zymosan particles/neutrophil in IMDM supplemented with 10% autologous serum and cultured at 37 °C for 12 h in a humidified, CO_2_-controlled incubator prior to labelling with annexin V and propidium iodide. The percentage of apoptotic cells is recorded. Each symbol represents the average value obtained in a separate experiment. Raw data were analyzed by Kruskal–Wallis test and multiple comparison post-hoc test. *p < 0.05; **p < 0.01.

Internalization of serum and/or IgG-opsonized particles induces PICD^6,8,27^. We compared induction of neutrophil apoptosis by iICs with a potent activator of PICD, opsonized zymosan. In contrast to iIC-induced apoptosis and consistent with previous reports^18,28^, PICD was PI3K- and Erk-independent (Fig. 3A, B). In keeping with previous observations^29,30^, the NADPH oxidase inhibitor diphenyleneiodonium (DPI) blocked both iIC-induced apoptosis and PICD (Fig. 3C, D). In contrast, inhibiting actin polymerization with cytochalasin B or latrunculin B inhibited PICD, whereas iIC-induced neutrophil apoptosis was not significantly reduced (Fig. 3C, D).

### Distinct internalization characteristics of iICs and IgG-opsonized beads

**Fig. 4:**
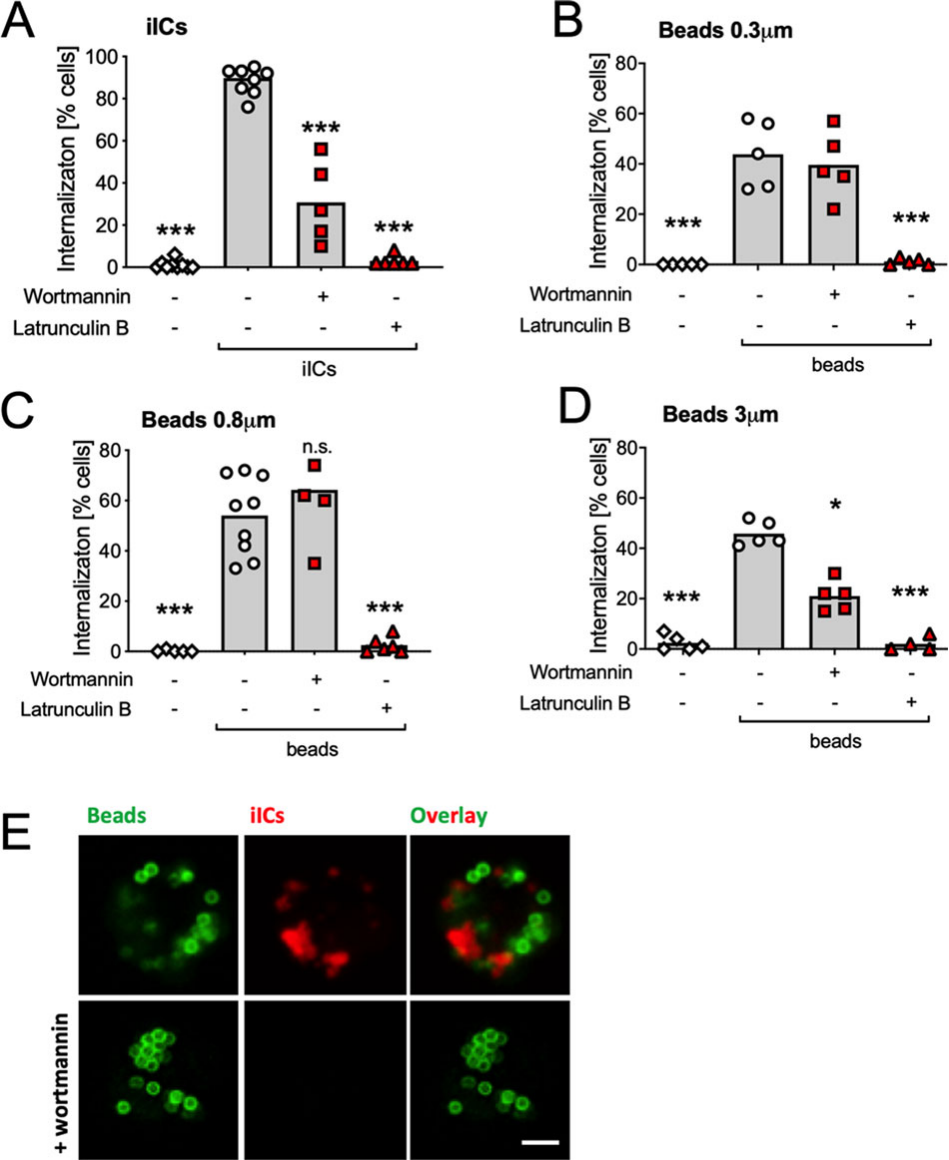
Internalization of iICs and beads is controlled by distinct pathways. Neutrophils were incubated with small molecule inhibitors or vehicle at 37 °C for 10 min as indicated to inhibit PI3Ks and actin polymerization, respectively, and stimulated by mixing with **A** 2 μg/ml iICs, **B–D** 5 IgG-opsonized beads/neutrophil of the indicated size or **E** a mixture of the iIC and 0.8 μm beads for a further 30 min. Neutrophils were then allowed to adhere to electrostatically charged glass slides on ice and fixed. Internalized and external iICs were labelled as detailed in ‘Materials and methods’. **A–D** The percentage of cells that had internalized particles was counted and plotted. Each symbol represents the average value obtained in a separate experiment. Raw data were analyzed by one-way ANOVA and multiple comparison post-hoc test; ***p < 0.001; *p < 0.05. **E** Representative confocal images taken with a Leica SP8 with 63× oil immersion objective of neutrophils that had or had not been pre-incubated with wortmannin prior to being simultaneously stimulated with pre-labelled beads (green) and iICs (red). Scale bar, 2 μm.

For further mechanistic insight into iIC-induced apoptosis, we compared the uptake of solid particles and iICs in more depth. To restrict neutrophil receptor involvement to a single receptor class, FcγR, we analyzed particle internalization with latex beads (0.3–3 μm diameter) that had been opsonized with polyclonal rabbit IgG in the absence of autologous serum (hereafter simply referred to as ‘beads’). Disrupting actin polymerization abrogated internalization of both iICs and beads. However, inhibiting PI3K blocked iIC internalization, but not uptake of 0.3 and 0.8 μm diameter beads (Fig. 4A–D; Fig. S3A, B), consistent with a requirement for PI3K in phagocytosis depending on particle size in other cells^31^.

These internalization experiments were performed 30 min after stimulation, while the induction of apoptosis occurs later (Fig. 1). We performed time courses, analyzing iIC internalization also after longer incubation times. Even 6 h after stimulation, when iIC-induced apoptosis was already obvious, iIC internalization by latrunculin B pre-treated neutrophils remained negligible (Fig. S3C). We concluded that iIC-induced neutrophil apoptosis, unlike PICD, can occur independently of iIC internalization.

Neutrophils efficiently phagocytosed 0.8 μm diameter beads, which once phagocytosed appeared closest in size to the vacuoles that contained internalized iICs (Fig. S3D). Further experiments were therefore carried out with these beads. Neutrophils stimulated simultaneously with iICs and beads efficiently internalized both. Inhibiting PI3K prior to co-stimulation with beads and iICs interfered with iIC internalization, but not bead phagocytosis (Fig. 4E), indicating that neutrophils use distinct mechanisms to internalize beads and iICs.

### iIC-induced neutrophil apoptosis and iIC internalization depend on FcγRII

**Fig. 5:**
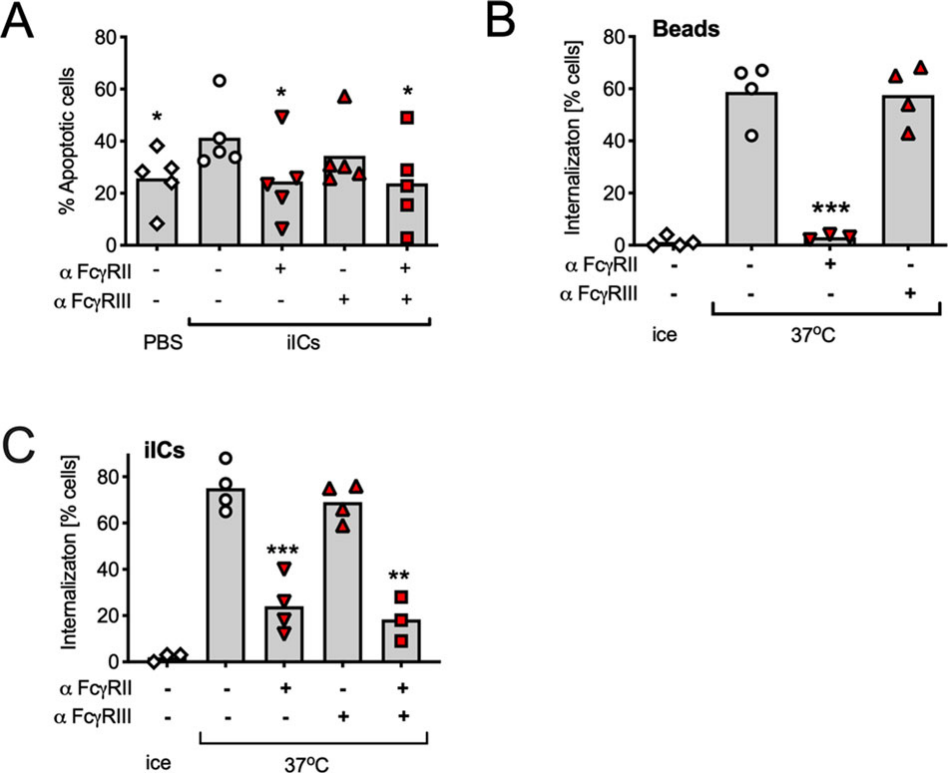
FcγR dependency of iIC-induced neutrophil apoptosis and iIC internalization. **A** Following incubation on ice with FcγR blocking antibodies (3G8, IV.3) or vehicle as indicated neutrophils were stimulated with 10 μg/ml iICs for analysis of neutrophil apoptosis after 12 h. Internalization of beads (**B**) and iICs (**C**) by neutrophils that had been incubated with blocking antibodies (3G8, AT10) or vehicle as indicated. Each symbol represents the average value obtained in a separate experiment. Data were subjected to analysis by one-way ANOVA with multiple comparisons post-hoc test; *p < 0.05; **p < 0.01; ***p < 0.001.

We used blocking antibodies to decipher the regulation of neutrophil apoptosis by iICs, identifying a major role FcγRII, without significant contribution of FcγRIII (Fig. 5A). Internalization experiments identified that blocking FcγRII inhibited internalization of both beads and iICs (Fig. 5B, C; Fig. S4A, B), contrasting with the differential involvement of PI3K.

### iICs signal through distinct cell surface receptors to trigger internal and external ROS production

**Fig. 6:**
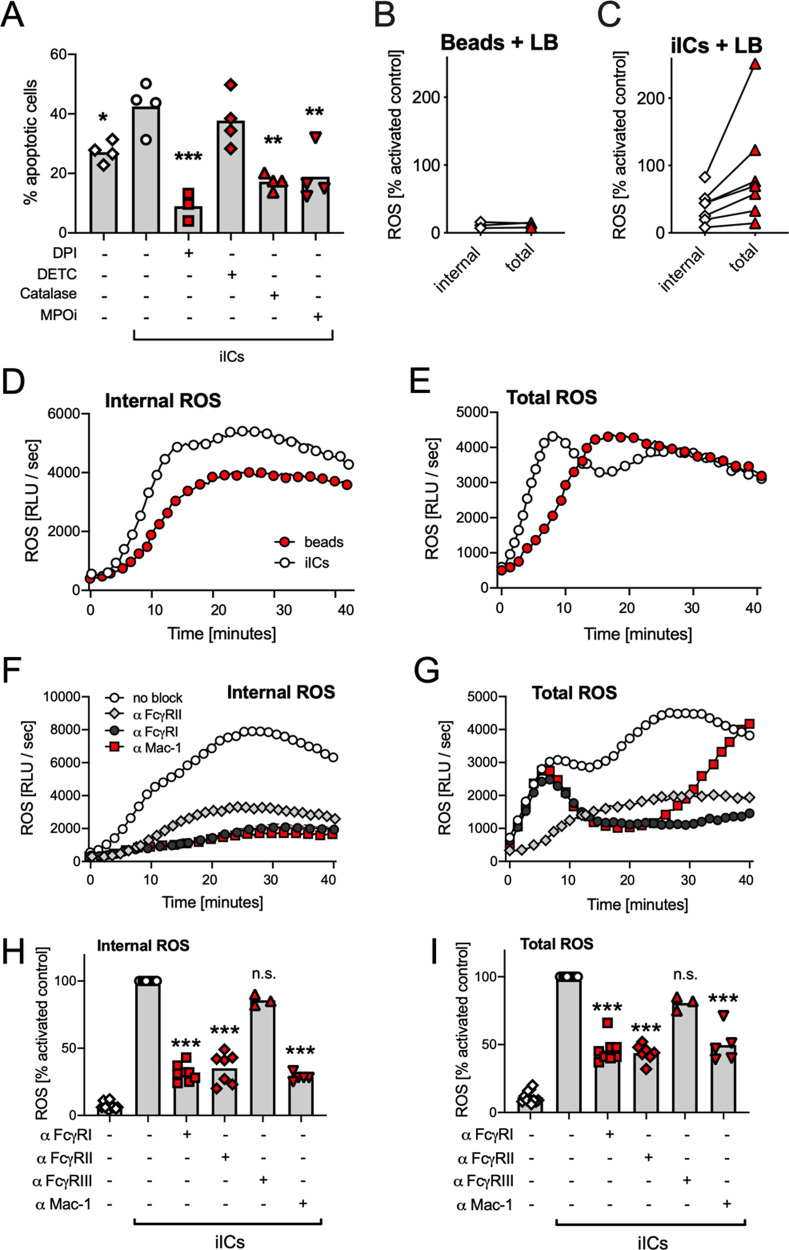
iIC-stimulated neutrophils produce FcμRII-dependent external ROS. **A** Following incubation with inhibitors, neutrophils were or were not stimulated with 10 μg/ml iICs for analysis of neutrophil apoptosis after 9 h. **B–I** Internal and total ROS production was analyzed in neutrophils that had been stimulated with 4 μg/ml iICs (**B**, **D-I**) or 25 beads/neutrophil (**C**) as detailed in ‘Materials and methods’. **B**, **C** Neutrophils were pre-treated for 10 min with inhibitor or vehicle as indicated prior to performing ROS assays. Internal (**D**, **F**, **H**) and total (**E**, **G**, **I**) ROS were analyzed with cells that had been pre-incubated for 30 min on ice with blocking antibodies (10.1, AT10, 3G8, ICRF44) or vehicle as indicated prior to stimulation with iICs. **D–G** Representative examples are shown; **H**, **I** Integrated total ROS production. **A-C**, **H, I** Each symbol represents the average value obtained in a separate experiment. **A**, **H**, **I** Raw data were analyzed by one-way ANOVA with multiple comparisons post-hoc test; *p < 0.05; **p < 0.01; ***p < 0.001; n.s. not significant.

In keeping with the observation that both iIC-induced apoptosis and PICD are ROS-dependent [Fig. 3C, D; (ref. ^28–30^)], inhibiting the NADPH oxidase or myeloperoxidase interfered with iIC-mediated induction of apoptosis. Incubating neutrophils with catalase, a scavenger of hydrogen peroxide that is not cell permeable also blocked the induction of iIC-induced apoptosis (Fig. 6A), suggesting a function of external ROS in this context. This contrasts with the predominant role of internal ROS reported in PICD^28^.

We further analyzed ROS produced in response to neutrophil stimulation by iICs or beads. Internal and total iIC and bead-induced ROS production was PI3K-dependent (Fig. S5A–C). Inhibiting bead phagocytosis by blocking actin polymerization abrogated bead-induced ROS (Fig. 6B). In contrast, inhibiting iIC internalization reduced internal ROS but resulted in increased external ROS (Fig. 6C). Indeed, when comparing production of internal and total ROS by neutrophils that had been stimulated with iICs or beads, we noticed an early peak of external ROS with iIC- but not bead-stimulated neutrophils (Fig. 6D, E for representative examples).

We used blocking antibodies to decipher the FcγR dependency of the internal and total ROS response to neutrophil stimulation with iICs. Unexpectedly, this identified that the initial peak of external ROS was entirely dependent upon FcγRII, whereas internal ROS production was mediated by FcγRI, Mac-1 and FcγRII (Fig. 6F–I; Fig. S5D, E). This suggested a dual role of FcγRII in mediating iIC internalization and generating external iIC-induced ROS.

### Neutrophils internalize iICs by receptor-dependent macropinocytosis

**Fig. 7:**
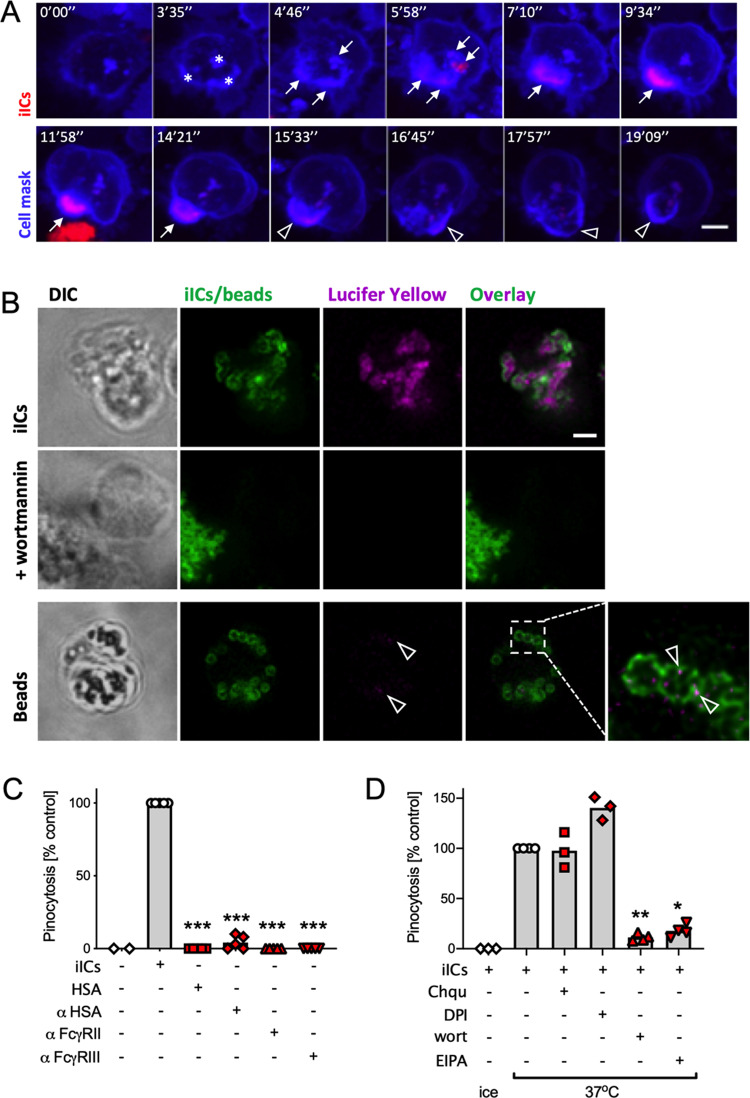
Neutrophils internalize iICs by macropinocytosis. **A** Neutrophils were incubated with a Cell Mask plasma membrane dye, allowed to adhere to glass coverslips and imaged using an Andor Revolution XDi spinning disc confocal microscope with 60× objective, acquiring roughly 2 images per minute. At time 0 cells were stimulated with fluorescently labelled iICs. Stills depict a single cell taken at the indicated timepoint are shown (see also Supplemental Movie 1). * denotes areas of abundant plasma membrane stain (likely ruffling) following stimulation; arrows identify internalized iICs. Arrowheads, loss of internalized iIC-associated fluorescence in the plasma membrane-derived vacuole. **B** Neutrophils were stimulated with 2 μg/ml iICs or 5 beads/neutrophil in the presence of the fluid phase marker lucifer yellow. Representative confocal images of neutrophils that had ingested iICs (top and middle) or beads (bottom) and in which PI3K had (middle) or had not (top, bottom) been inhibited. Arrowheads, lucifer yellow internalization that accompanied phagocytosis. **A**, **B** Scale bars, 2 μm. Pinocytosis was assessed by uptake of lucifer yellow with neutrophils (**C**) that had been stimulated with vehicle, iICs, components thereof (HSA and anti-HSA) or FcγR blocking antibodies (AT10, 3G8) or (**D**) that had been pre-incubated with small molecule inhibitors as indicated (Chqu chloroquine, DPI diphenyleneiodonium, wort wortmannin, EIPA ethylisopropylamiloride) prior to stimulation with iICs. **C**, **D** Cells that had undergone macropinocytosis (i.e., taken up lucifer yellow) were counted. Each symbol represents the average value obtained in a separate experiment. Raw data were subjected to analysis by one-way ANOVA with multi comparison post-hoc test; *p < 0.05; **p < 0.01; ***p < 0.001.

Our results suggested that iICs were internalized by a process other than phagocytosis. We used time-lapse imaging of neutrophils in which the plasma membrane had been dyed with cell mask to test whether iICs entered an intracellular compartment that was derived from the plasma membrane. We noted rapid uptake of fluorescently labelled iICs at several distinct locations within an area of bright cell mask suggestive of localized plasma membrane accumulation (Fig. 7A, asterisks). The distinct foci of internalized iICs subsequently merged into a vacuole, which was initially characterized by bright iIC-associated fluorescence, fading rapidly, leaving behind the circular, cell mask-positive vacuole (Fig. 7A and Supplemental Movie 1).

The prominent plasma membrane structures preceding iIC internalization (Fig. 7A) were suggestive of membrane ruffling. We therefore asked whether the internalization process was due to macropinocytosis, an endocytic process that is initiated by the formation of PI3K-dependent, circular membrane ruffles that initiate fluid internalization^32–34^. Internalization experiments performed in media containing lucifer yellow (Fig. 7B) or FITC-dextran (Fig. S6) identified iIC-lined macropinosomes filled with fluid phase markers; internalization of the fluid phase markers was PI3K-dependent. In contrast, fluid uptake as defined by lucifer yellow signal was minimal in neutrophils phagocytosing beads (Fig. 7B, arrowheads). Neutrophil macropinocytosis did not occur constitutively, but was dependent upon the presence of iICs. Neither iIC constituents, HSA, anti-HSA, nor FcγRII/III blocking antibodies alone were able to induce macropinocytosis (Fig. 7C). Macropinocytosis was inhibited by pre-treating neutrophils with the pinocytosis inhibitor EIPA, but not with an autophagy inhibitor (chloroquine) or DPI (Fig. 7D).

### Degradation of iICs

**Fig. 8:**
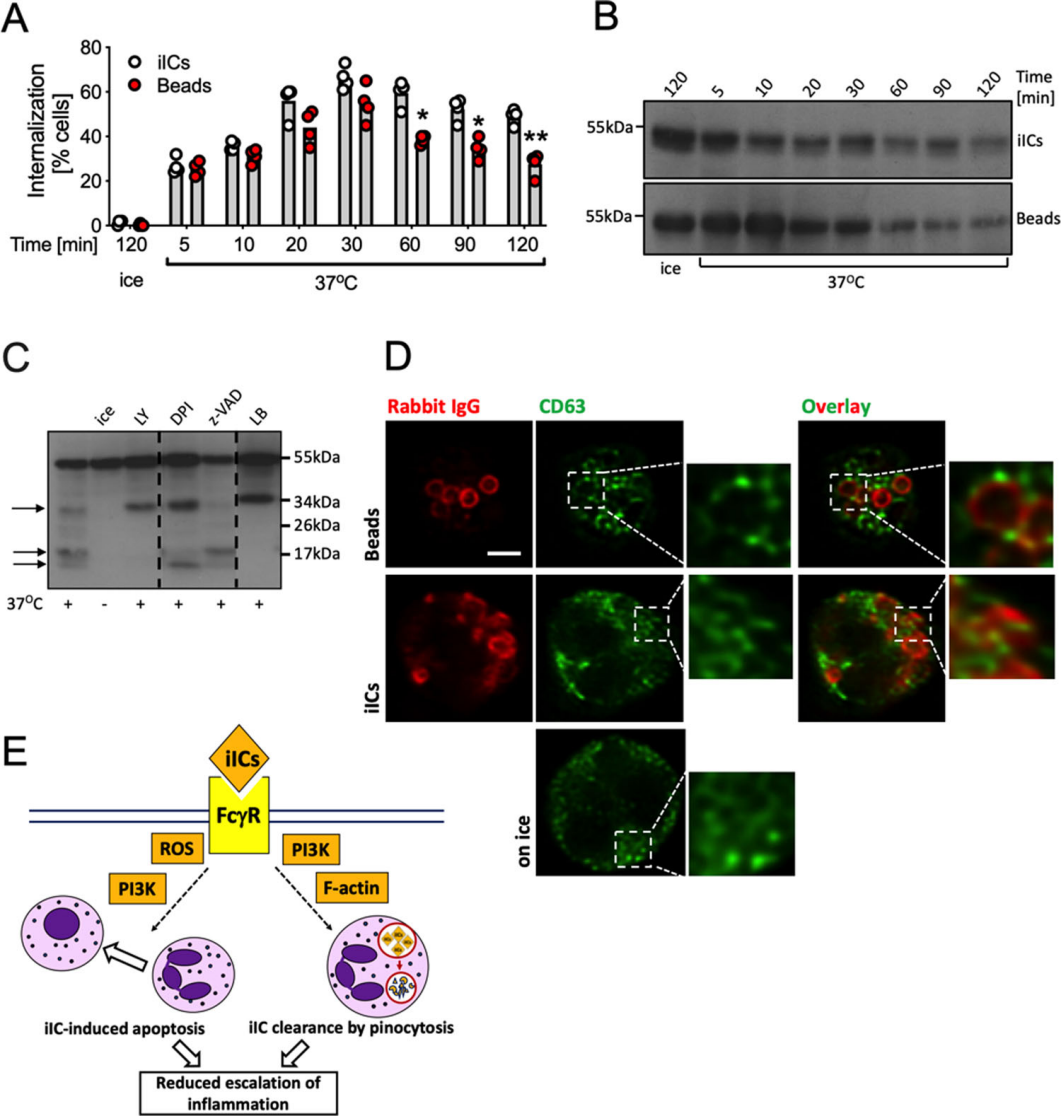
Internalized iICs are subject to rapid degradation. **A** Suspension neutrophils at 37 °C or on ice were stimulated with 2 μg/ml iICs or 5 IgG-opsonized beads/neutrophil for the indicated times, allowed to attach to glass slides on ice, fixed, and labelled for external and total particles. Cells containing internalized particles are plotted. Each symbol represents the average value obtained in a separate experiment. Data were analyzed by two-way ANOVA with multiple comparisons post-hoc test; *p < 0.05; **p < 0.01. Neutrophils were stimulated with 2 μg/ml iICs for the indicated time at 37 °C or on ice (**B**), or after being pre-incubated with inhibitors or vehicle as indicated to inhibit PI3K, ROS production, caspases and actin polymerization (**C**; LY, LY294002; DPI diphenyleneiodonium, z-VAD z-VAD-FMK, LB latrunculin B, respectively). Cell lysates were subjected to SDS-PAGE, transferred to PVDF membrane and probed for the rabbit IgG heavy chain. Representative blots are shown from ≥3 separately performed experiments. **D** Neutrophils were or were not stimulated with iICs or IgG-opsonized beads as in (**A**), and allowed to adhere to glass slides prior to labelling CD63 and rabbit IgG. Samples were viewed on a Leica SP8 confocal microscope with 63× objective. Representative examples are shown. Scale bar, 2 μm. **E** Schematic diagram illustrating the two concomitant iIC-induced neutrophil anti-inflammatory pathways, iIC macropinocytosis and iIC-induced neutrophil apoptosis.

To examine the kinetics of iIC internalization, we performed time courses, labelling internalized iICs or beads after fixation. Internalization was observed as early as 5 min after stimulation, with peak uptake recorded at 30 min (Fig. 8A). At later timepoints we noted reduced fluorescence, suggesting that the internalized iICs were being degraded. Western blotting confirmed proteolysis of the IgG heavy chain associated with both iICs and beads (Fig. 8B). On overexposed blots IgG degradation products were clearly visible (Fig. 8C, arrows). We noted a degradation band ~34 kDa, and a doublet ~17 kDa with cells that had been stimulated with iICs at 37 °C. Blocking iIC internalization by inhibiting PI3K or actin polymerization caused the appearance of a prominent ~34 kDa band, suggestive of degradation even of non-internalized iICs by a surface protease. With neutrophils treated with the NADPH oxidase inhibitor DPI or the pan-caspase inhibitor z-VAD-FMK, differential banding patterns and smaller degradation products were observed. iIC degradation was inhibited in cells that had been incubated with DFP, a potent inhibitor of serine proteases, but not by E64, an inhibitor of cysteine proteases (Fig. S7A); DFP treatment, but not E64 also inhibited the induction of apoptosis (Fig. S7B). Together our observations suggested that iIC degradation occurred in a stepwise fashion involving several proteases.

Neutrophils are characterized by specialized granules that contain cytotoxic components and proteolytic enzymes. In phagocytosis, granules fuse with the phagosome, emptying their cargo into it for intracellular killing. We stained neutrophils that had or had not been allowed to internalize beads or iICs with CD63, a marker of azurophil granules^35^. CD63 localized to distinct speckles in unstimulated neutrophils, which reorganized in cells that had ingested beads such that CD63-positive spots surrounded beads. In contrast, CD63 signal did not neatly surround the vacuoles containing iICs (Fig. 8D), but instead showed some elongated structures which overlapped with iIC-containing vacuoles.

Altogether, our results demonstrate two separate FcγR-dependent pathways in iIC-stimulated neutrophils, (i) the induction of apoptosis in a PI3K- and ROS-dependent, but actin-independent fashion, and (ii) the actin- and PI3K-dependent internalization by macropinocytosis and subsequent digestion of iICs (Fig. 8E).

## Discussion

Prompted by the observation that iIC-induced neutrophil apoptosis occurs concomitantly with iIC internalization, we compared iIC-induced neutrophil apoptosis and PICD. Our findings suggest that these 2 specialized types of apoptosis are regulated by different signalling cascades, with iIC-induced neutrophil apoptosis, but not PICD promoted by PI3K and Erk [Fig. 3 (ref. ^18,28^)].

A second difference between the two cell death processes is that PICD, but not iIC-induced neutrophil apoptosis depends upon particle internalization. We found that iICs and IgG-opsonized beads are internalized by two different mechanisms which can take place simultaneously within the same cell (Fig. 4E). iIC internalization occurs by class I PI3K-dependent macropinocytosis rather than phagocytosis (Fig. 7B), with iIC internalization sensitive to pan-PI3K inhibition (Fig. S2C) at inhibitor concentrations previously described for macrophage pinocytosis^32^. Macropinocytosis is a comparatively poorly defined process that is initiated by circular ruffles, which close around extracellular fluid, engulfing it. Unlike constitutive macropinocytosis, which professional antigen presenting cells (e.g. dendritic cells, macrophages) employ to sample extracellular antigen for presentation of peptides^36–39^, our data demonstrate that neutrophil macropinocytosis of iICs is FcγR mediated (Fig. 5C). FcγR stimulation during phagocytosis was previously shown to induce concurrent receptor-mediated focal pinocytosis in the neutrophil^40^ in a process that correlated with the secretion of primary granules. We also noticed a small amount of fluid phase marker uptake by neutrophils that were phagocytosing beads (arrowheads in Fig. 7B). Unlike the localized pinocytosis accompanying phagocytosis^40^, iIC-induced macropinocytosis was dependent upon the intact actin cytoskeleton.

Both iIC-induced neutrophil apoptosis and PICD share their dependency on ROS production (Fig. 6A). A closer examination identified that ROS induced by beads and iICs exhibited key differences. We observed an early, FcγRII-mediated peak of external ROS with iIC-stimulated neutrophils, and while inhibiting bead internalization abolished PICD-associated ROS production, inhibition of iIC internalization induced a switch from internal to external ROS. Interestingly, incubating neutrophils with an FcγRII blocking antibody inhibited iIC internalization and the induction of apoptosis (Fig. 5A, C), whereas blocking iIC internalization by inhibiting actin polymerization was not sufficient to interfere with the induction of apoptosis (Fig. 3C). The observation that the cell-impermeable ROS scavenger catalase inhibits iIC-induced apoptosis (Fig. 6A) suggests that the initial wave of external ROS that occurs upon stimulation of neutrophils with iICs (Fig. 6D–G) may be sufficient to induce apoptosis. Similarly, the increased external ROS generated upon iIC stimulation of neutrophils in which actin polymerization has been inhibited (Fig. 6C) are likely to promote the induction of neutrophil apoptosis even in the absence of iIC internalization.

A second unexpected feature of iIC-induced ROS production was the cell surface receptor involvement. FcγRII ligation was found to be responsible for the initial wave of external ROS, while the subsequently produced internal ROS were largely due to FcγRI and Mac-1 (Fig. 6F, G). This suggests that low levels of FcγRI on circulating human neutrophils are sufficient to make a marked contribution to the generation of ROS released into macropinosomes of neutrophils stimulated with iICs. Mac-1 is an integrin that has been shown to bind to multiple ligands, including ICAM1, fibrinogen and complement components^41–43^. In our experiments iICs were prepared from lyophilized HSA and affinity purified polyclonal rabbit anti-HSA IgG. Internalization occurred in the absence of serum, suggesting that involvement of complement in this context is unlikely. However, extensive cross-talk between β2 integrins and FcγRs has previously been documented by many groups. With immobilized ICs, for example, FcγRs were found to be sufficient for initial interaction of neutrophils with immobilized ICs, but Mac-1 activity was required for sustained binding^44,45^.

Both internalization mechanisms we have examined here, FcγR-mediated phagocytosis and macropinocytosis, lead to the rapid degradation of internalized IgG (Fig. 8; Fig. S6). Our results suggest that iICs are degraded in a stepwise fashion by several proteases and that some extracellular digestion of IgG occurs prior to internalization. This might be mediated by proteinase-3, a plasma membrane-localized serine protease that colocalises with FcγRIII^46,47^, while intracellular serine proteases, e.g., those stored in azurophil granules may be responsible for intracellular iIC degradation. While we were unable to identify individual proteases involved, the powerful serine protease inhibitor DFP interfered with iIC degradation as well as neutrophil apoptosis (Fig. S6), consistent with previous reports which showed that DFP inhibits constitutive neutrophil apoptosis^48,49^.

In the context of iIC-induced neutrophilic inflammation, our observation of iIC degradation following their internalization by neutrophils suggests that neutrophils make an important contribution to the clearance of iICs. Given that several studies have described ICs localized to vacuoles in human patient neutrophils [e.g. (ref. ^50–53^)], we propose that the mechanism elucidated here is of pathophysiological relevance. Circulating iICs are highly prone to being deposited on host tissue surfaces where they potently stimulate neutrophilic inflammation resulting in host tissue injury as happens in autoimmune diseases, including rheumatoid arthritis. The fact that neutrophils are involved in clearance of these large ICs suggests hitherto unappreciated anti-inflammatory function aimed at preventing host tissue injury. Intriguingly, iICs are not only degraded by neutrophils, but independently and concurrently also induce neutrophil apoptosis^18,30^. It is well-documented that the induction of neutrophil apoptosis coincides with the release of ‘find-me’ and ‘eat-me’ signals^54^ and ultimately efferocytosis by tissue macrophages, dampening inflammation and promoting resolution and the release of pro-resolving mediators^4,55^. These iIC-induced neutrophil anti-inflammatory functions essentially operate as a safeguard, promoting iIC clearance, as well as neutrophil clearance by apoptosis. In this way neutrophils contribute to attenuating the escalation of iIC-induced inflammation.

## Supplementary information

Supplementary figure legends

Supplementary tables

Figure S1

Figure S2

Figure S3

Figure S4

Figure S5

Figure S6

Figure S7

movie S1

## Data Availability

All data generate or analyzed during this study are included in this published article and its supplementary information files.
