# Supplementary material for: Immune complex-induced apoptosis and concurrent immune complex clearance are anti-inflammatory neutrophil functions
Source: Cell Death Dis. 2021 Mar 19;12(4):296. doi: 10.1038/s41419-021-03528-8 (PMC7979711; doi:10.1038/s41419-021-03528-8)
Supplement: Supplementary file 1 — Supplementary figure legends [file 41419_2021_3528_MOESM1_ESM.docx]

**Supplementary Figure Legends**

This article contains 7 supplementary figures, 1 video file and 2 supplementary tables.

**Figure S1. Analysis of iIC-induced apoptosis.** (A) Gating for the analysis of neutrophil apoptosis by flow cytometry. Debris and doublets were excluded prior to analysing cells for propidium iodide and annexin V-FITC. Unstained controls of cells that were or were not stimulated with iICs at t=0 hrs are included to show that increased autofluorescence is a result of iIC-stimulation; this is likely due to the uptake of phenol red-containing culture medium (see also Figs 7, S6). Double negative cells were scored viable, PI^-^ AV^+^ cells apoptotic and PI^+^ cells as having lost their membrane integrity. Examples of neutrophils that were or were not stimulated with iICs for t=12 hrs are included to showcase all of these populations. Neutrophils incubated with a cytotoxic concentration of a protease inhibitor, Pefabloc (Pefa), also lost their membrane integrity. (B, C) Neutrophils were pre-incubated with small molecule inhibitor indicated (z-VAD, z-VAD-FMK, pan-caspase inhibitor) at 37°C for 10 minutes, stimulated with 10μg/ml iICs (HSA anti-HSA) in IMDM supplemented with 10% autologous serum and cultured at 37°C. After 6 hrs cells (B) were stained with annexin V and propidium iodide and analysed for the induction of apoptosis by flow cytometry; (C) a cytospin was prepared and cytoplasm and nuclei stained with Diff Quick. Boxed cell is shown enlarged. Scale bar, 10μm. (D) Gating for the analysis of activation of caspase-3 by flow cytometry. Debris and doublets were excluded prior to analysing neutrophils that had been stimulated, cultured, fixed and subjected to intracellular staining with an AF488-conjugated monoclonal directed against cleaved human caspase-3. (E) Cleaved caspase-3 was detected by flow cytometry in neutrophils that had been pre-incubated with inhibitors as indicated prior to being stimulated with iICs or its vehicle (PBS) as indicated and cultured for 0 or 7 hours. Raw data were subjected to analysis by 1-way ANOVA and multi comparisons post-hoc test, comparing all conditions to iIC-stimulated neutrophils. ** p<0.01. (F) Detection of total and cleaved caspase-3 as well as a loading control (RhoA) by Western blotting. Prominent IgG degradation products (asterisks) in cells that were stimulated with iICs irrespective of caspase inhibition obscure the smaller activated caspase-3 band in iIC stimulated cells in the absence of caspase inhibition since cleaved caspase-3 was detected here with a rabbit polyclonal. A representative blot is shown. See Figs 8 and S7 for additional information on iIC degradation by neutrophils.

**Figure S2. Inhibiting PI3K interferes with internalization of iICs by neutrophils.** (A) Gating strategy for analysis of iIC internalization by flow cytometry. Debris and doublets were excluded prior to analysis of cells stimulated with AF-488 labelled iICs that had been incubated with a AF-647 labelled secondary antibody after 30 minutes at 37°C (on ice for negative control). AF-488^-^ AF-647^-^ cells were scored negative, AF-488^+^ AF-647^-^ cells were scored as having internalised iICs and AF-488^+^ AF-647^+^ cells as having bound (but not internalized) iICs. Please note that the amount of iICs bound / internalized is variable, making for spread out populations; this scoring system counts cells that internalized and bound iICs as ‘bound’. For single stains, cells were stimulated with AF-488 or AF-647-prelabelled iICs. (B) Cells were or were not incubated with 10μM of LY294002 (LY) for 10 minutes at 37°C prior to being stimulated with 10μg/ml iICs and cultured at 37°C in IMDM in the presence of 10% autologous serum. 6 hrs later cells were spun onto slides, and internalised and external iICs labelled with nuclear counterstain (DAPI). A representative image is shown. LY294002 reduced the amount of iICs internalised, while not abolishing internalization. Scale bar, 5μm. (C, D) Neutrophils were incubated with pan-PI3K inhibitors at the indicated concentrations, and stimulated with 10μg/ml iICs on ice or at 37°C for 30 minutes prior to analysis as detailed in (A). Cells that had internalized iICs are plotted in (C), while cells that had bound iICs are plotted in (D). Each symbol represents the average value obtained in a separate experiment. Data analysis was by 1-way ANOVA and multiple comparison post-hoc test. * p<0.05; ** p<0.01, *** p<0.001.

**Figure S3. Actin polymerization is required for iIC internalization, but not for the induction of iIC-induced neutrophil apoptosis.** (A, B) Neutrophils were incubated with inhibitors as indicated and stimulated with 10μg/ml iICs on ice or at 37°C for 30 minutes prior to analysis of iIC internalization by flow cytometry. Cells that had internalised iICs are plotted in (A), while cells that had bound iICs are plotted in (B). Each symbol represents the average value obtained in a separate experiment. Data analysis was by 1-way ANOVA and multiple comparison post-hoc test. *** p<0.001. (C) Cells were or were not pre-treated with inhibitors as indicated prior to being stimulated with 10μg/ml iICs and cultured at 37°C in IMDM in the presence of 10% autologous serum. At the indicated times, cytospins were prepared, internalized and external iICs were labelled, with a nuclear counterstain. DFP- and DPI- treated controls were included which internalized iICs but did not undergo apoptosis. Scale bar, 6μm. (D) Neutrophils were stimulated with iICs or IgG-opsonized beads of the indicated sizes, and internalised and attached particles stained as detailed in Materials and Methods. Representative images are shown. Scale bar, 2μm.

**Figure S4. iIC-internalization is mediated by FcγRII.** Neutrophils were incubated with blocking antibodies for 30 minutes on ice as indicated prior to (A) stimulation with iICs and analysis of iIC internalization as in Fig 4C or (B) stimulation with 10μg/ml iICs and being cultured at 37°C in IMDM in the presence of 10% autologous serum for 6 hrs. Cytospins were prepared for labelling of internalized and attached iICs. Representative images are shown. Scale bar, 6μm. (A) Each symbol represents the average value obtained in a separate experiment. Data analysis was by 1-way ANOVA and multiple comparison post-hoc test. ** p<0.01; *** p<0.001.

**Figure S5. Characterization of ROS production by iIC-stimulated neutrophils.** Neutrophils were incubated with (A-C) inhibitors for 10 minutes at 37°C or (D, E) blocking antibodies for 30 minutes on ice as indicated prior to (A-E) stimulation with iICs or IgG-opsonized beads as indicated and analysis of internal and total ROS production as detailed in materials and methods. Each symbol represents the average value obtained in a separate experiment. Raw data were (A) subjected to pairwise comparison by Mann Whitney test or (D, E) analysed by 1-way ANOVA with multiple comparisons post-hoc test; *** p<0.001.

**Figure S6. Neutrophils internalize iICs by macropinocytosis.** Neutrophils were stimulated with 2 μg/ml iICs or 5 beads/ neutrophil in the presence of a fluid phase marker (FITC- dextran). Representative confocal images of neutrophils that had ingested iICs (top and middle) or beads (bottom) are shown. Scale bar, 2μm.

**Figure S7. iICs are rapidly degraded following internalization.** (A) Neutrophils were treated with inhibitors or their vehicle as indicated prior to stimulation with 2μg/ml iICs and incubation on ice or at 37°C for 90 minutes. Lysates were prepared and processed for Western blotting detecting the IgG heavy chain. (B) Following inhibitor treatment, neutrophils were stimulated with 10μg/ml iICs and cultured at 37°C in IMDM in the presence of 10% autologous serum for 6 hrs for analysis of the induction of apoptosis. Each symbol represents the average value obtained in a separate experiment. Raw data were analysed by 1-way ANOVA with multiple comparisons post-hoc test; * p<0.05.

**Movie S1. Internalization of iICs.** Neutrophils that had been incubated with a Cell Mask plasma membrane dye (pseudocoloured blue) were allowed to adhere to glass coverslips and imaged using an Andor Revolution XDi spinning disc confocal microscope with 60x objective, acquiring roughly 2 images per minute. At time 0 cells were stimulated with fluorescently labelled iICs (red). This movie shows a slightly larger field of view than the stills shows in Fig 7A with three neutrophils that are rapidly internalizing iICs. The cell shown in Fig 7A is indicated by a white arrow. Membrane ruffling is indicated by *, foci of iIC internalization by arrows, and their condensation into vacuoles by arrowheads.
