# Supplementary material for: Immune complex-induced apoptosis and concurrent immune complex clearance are anti-inflammatory neutrophil functions
Source: Cell Death Dis. 2021 Mar 19;12(4):296. doi: 10.1038/s41419-021-03528-8 (PMC7979711; doi:10.1038/s41419-021-03528-8)
Supplement: Supplementary file 2 — Supplementary tables [file 41419_2021_3528_MOESM2_ESM.docx]

**Supplementary Tables**

*Table 1. Inhibitors used for this study*

| **Inhibitor** | **Target** | **Working concentration^1^** | **Manufacturer** | **Identifier** |
| --- | --- | --- | --- | --- |
| **LY294002 ^2^** | Pan-PI3K | 10 µM | Synkinase | SYN-1108-M001 |
| **Wortmannin ^2^** | Pan-PI3K | 100 nM | Sigma | W1628 |
| **FR180204** | Erk | 10 µM | Selleckchem | S7524 |
| **Z-VAD-FMK** | Pan-Caspase | 100 µM | Cayman  Bachem | A12373  4027403 |
| **QVD** | Pan-Caspase | 10 µM | R&D Systems | OPH001-01M |
| **Roscovitine** | CDK2/7 | 20 µM | Cayman | 10009569 |
| **Cytochalasin B** | Actin | 10 µM | PanReac AppliChem | A7657 |
| **Latrunculin B** | Actin | 10 µM | Calbiochem | CALB428020-1 |
| **DFP (Diisopropyl**  **Fluorophosphate)** | Serine proteases | 7 mM | Sigma | D0879 |
| **Pefabloc SC (AEBSF)** | Serine proteases | 2.5 mM  (cytotoxic) | Sigma | 76307 |
| **E64** | Cysteine proteases | 2.5 µM | Sigma | E3132 |
| **DPI** | NADPH oxidase | 10 µM | Cayman | 81050 |
| **DETC**  **(diethyldithiocarbamate)** | Superoxide dismutase | 10 µM | Sigma | D93503 |
| **Catalase** | Hydrogen peroxide | 25 µg/ml | Sigma | C1345 |
| **MPO inhibitor I** | Myeloperoxidase | 0.5 mM | Sigma | 475944 |
| **EIPA** | Pinocytosis | 50 µM | Sigma | A3085 |

^1^ Unless indicated otherwise, inhibitors were not cytotoxic at the concentrations and times employed*.*

^2^ Since wortmannin is short-lived in aqueous solutions, LY294002 was used for prolonged PI3K inhibition*.*

*Table 2. Antibodies used for this study*

| **Antibody** | **Clone (monoclonals) /**  **Isotype (blocking Abs)** | **Working concentration** | **Source** | **Identifier** |
| --- | --- | --- | --- | --- |
| Anti-FcγRIII | 3G8 / IgG1 | 5 µg/ml | (in-house) |  |
| Anti-FcγRII F(ab’)_2_ | AT10 / IgG1 | 5 µg/ml | Martin Glennie |  |
| Anti-FcγRII | IV.3 / IgG2b | 5 µg/ml | (in-house) |  |
| Anti-FcγRI | 10.1 / IgG1 | 10 µg/ml | Biolegend | 305002 |
| Anti-Mac1 | ICRF44 / IgG1 | 12.8 µg/ml | Nancy Hogg |  |
| Anti-CD63 | H5C6 | 1:300 | Biolegend | 353013 |
| Anti-Caspase 3 | 19 | 1:1000 | BD Biosciences | 610322 |
| Anti-human cleaved caspase 3-AF488 | D3E9 | 1:50 | Cell Signaling Technology | 9603 |
| Anti-cleaved caspase 3 |  | 1:1000 | Cell Signaling Technology | 9661 |
| Anti-Gelsolin | CPTC-Gelsolin2 | 1:500 | Biolegend | 866501 |
| RhoA | 26C4 | 1:1000 | Santa Cruz Biotechnology | sc-418 |
| Alexafluor 488 goat anti-rabbit IgG (H+L) |  | 1:400 | Invitrogen | A11008 |
| Alexafluor 488 goat anti-mouse IgG (H+L) |  | 1:400 | Invitrogen | A11029 |
| Alexafluor 568 goat anti-rabbit IgG (H+L) |  | 1:400 | Invitrogen | A11011 |
| Alexafluor 647 goat anti-rabbit IgG (H+L) |  | 1:400 | Biolegend | 406414 |
| Rabbit polyclonal IgG |  | (see Materials and Methods ) | Sigma | I8140 |
| Rabbit Anti-HSA (Polyclonal IgG) |  | (see Materials and Methods ) | Sigma | A0433 |
| HRP-conjugated anti-rabbit secondary antibody |  | 1:3000 | BioRad | 170-6515 |
| HRP-conjugated anti-mouse secondary antibody |  | 1:2000 | BioRad | 170-6516 |
