# Supplementary figures and images for: Immune complex-induced apoptosis and concurrent immune complex clearance are anti-inflammatory neutrophil functions
Source: Cell Death Dis. 2021 Mar 19;12(4):296. doi: 10.1038/s41419-021-03528-8 (PMC7979711; doi:10.1038/s41419-021-03528-8)

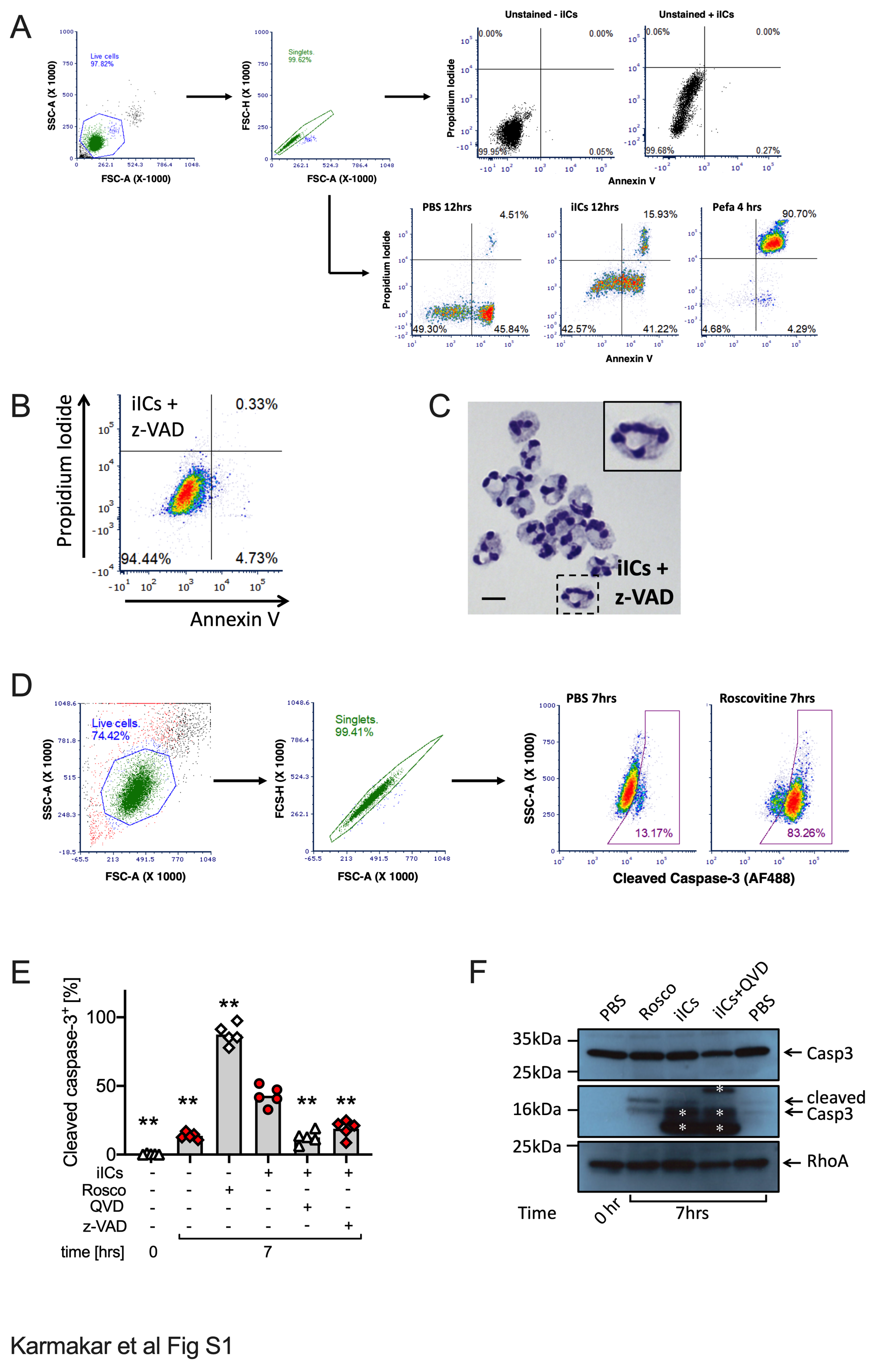

Supplement: Supplementary file 3 — Figure S1 [file 41419_2021_3528_MOESM3_ESM.png]

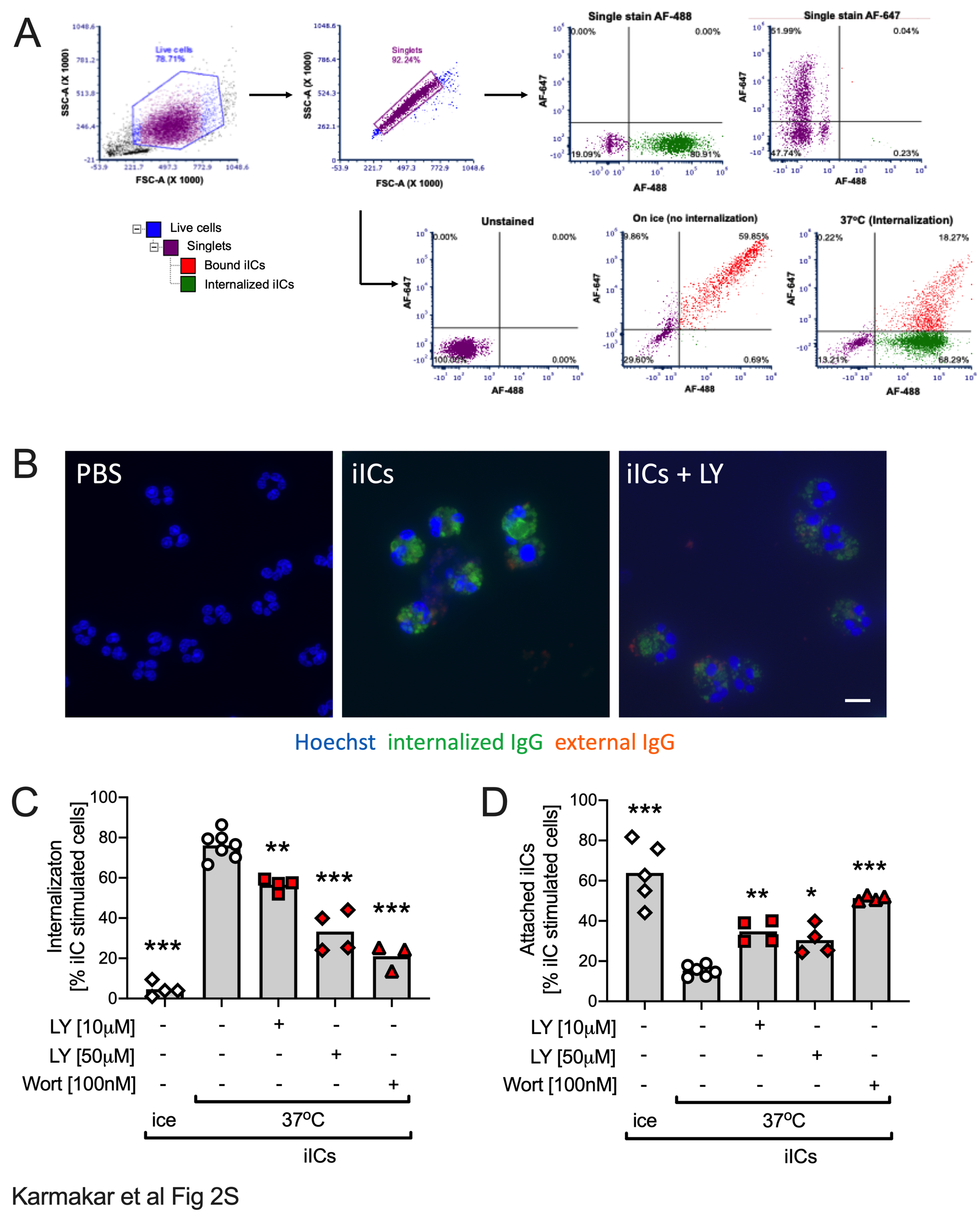

Supplement: Supplementary file 4 — Figure S2 [file 41419_2021_3528_MOESM4_ESM.png]

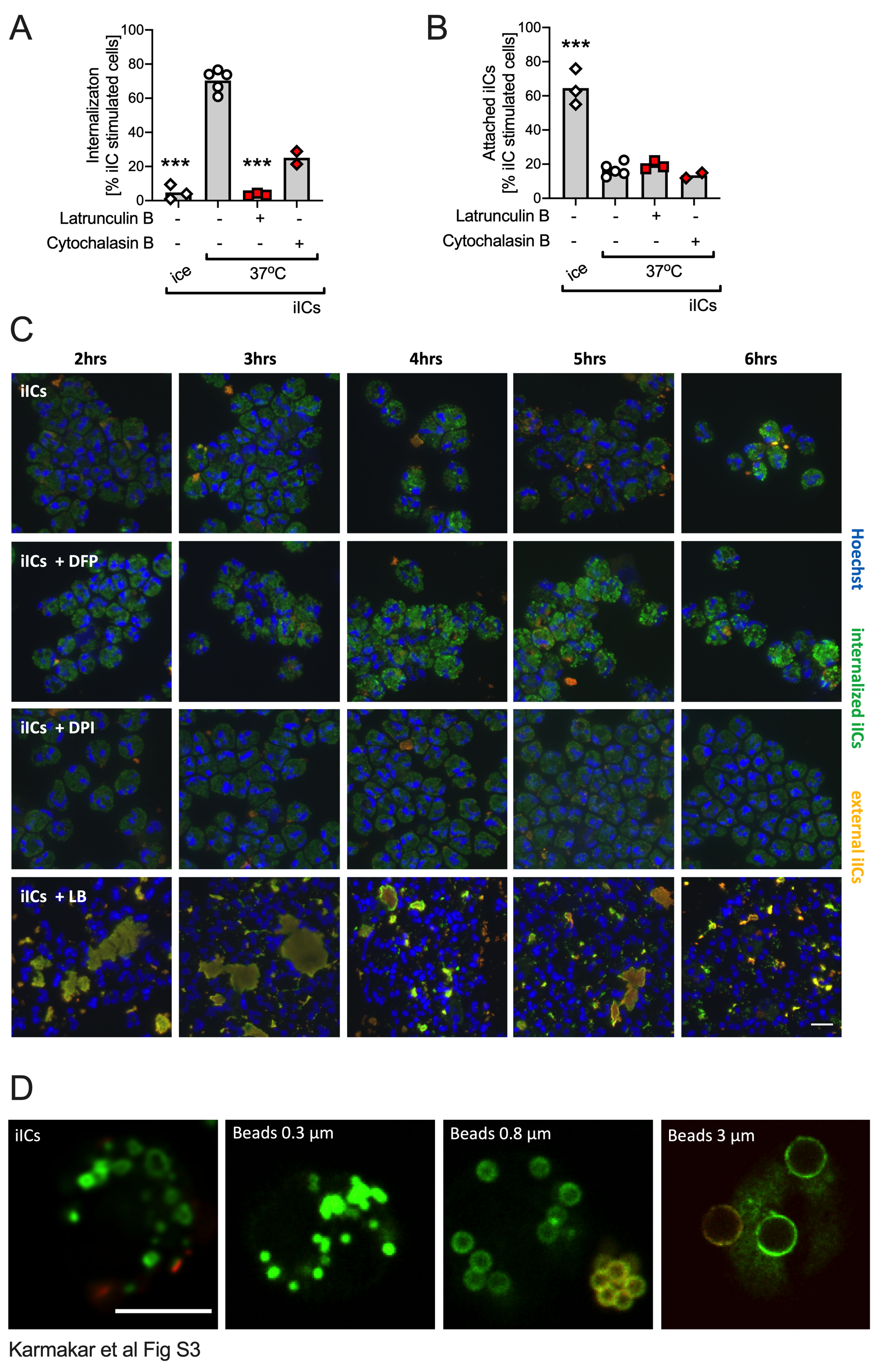

Supplement: Supplementary file 5 — Figure S3 [file 41419_2021_3528_MOESM5_ESM.png]

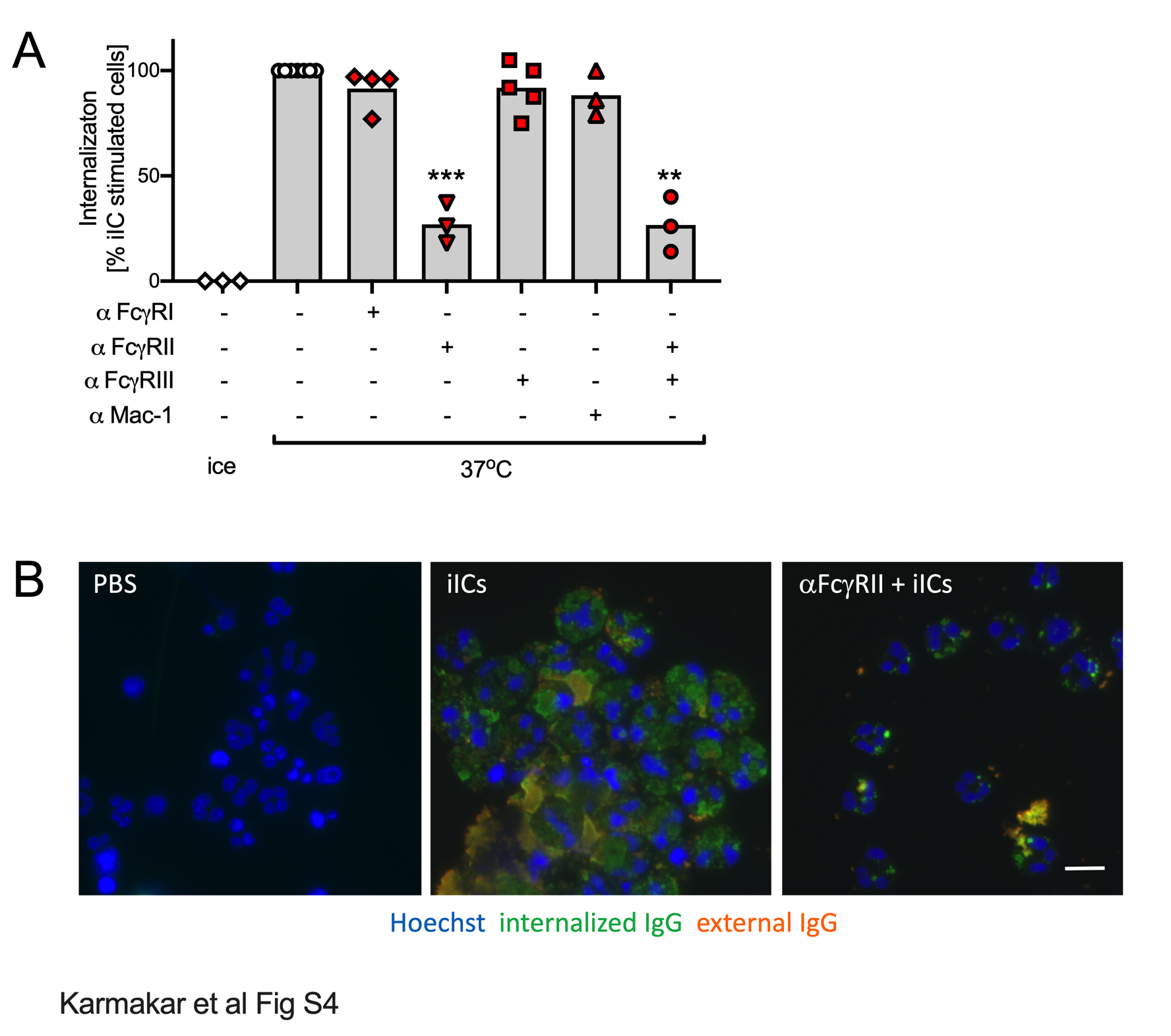

Supplement: Supplementary file 6 — Figure S4 [file 41419_2021_3528_MOESM6_ESM.png]

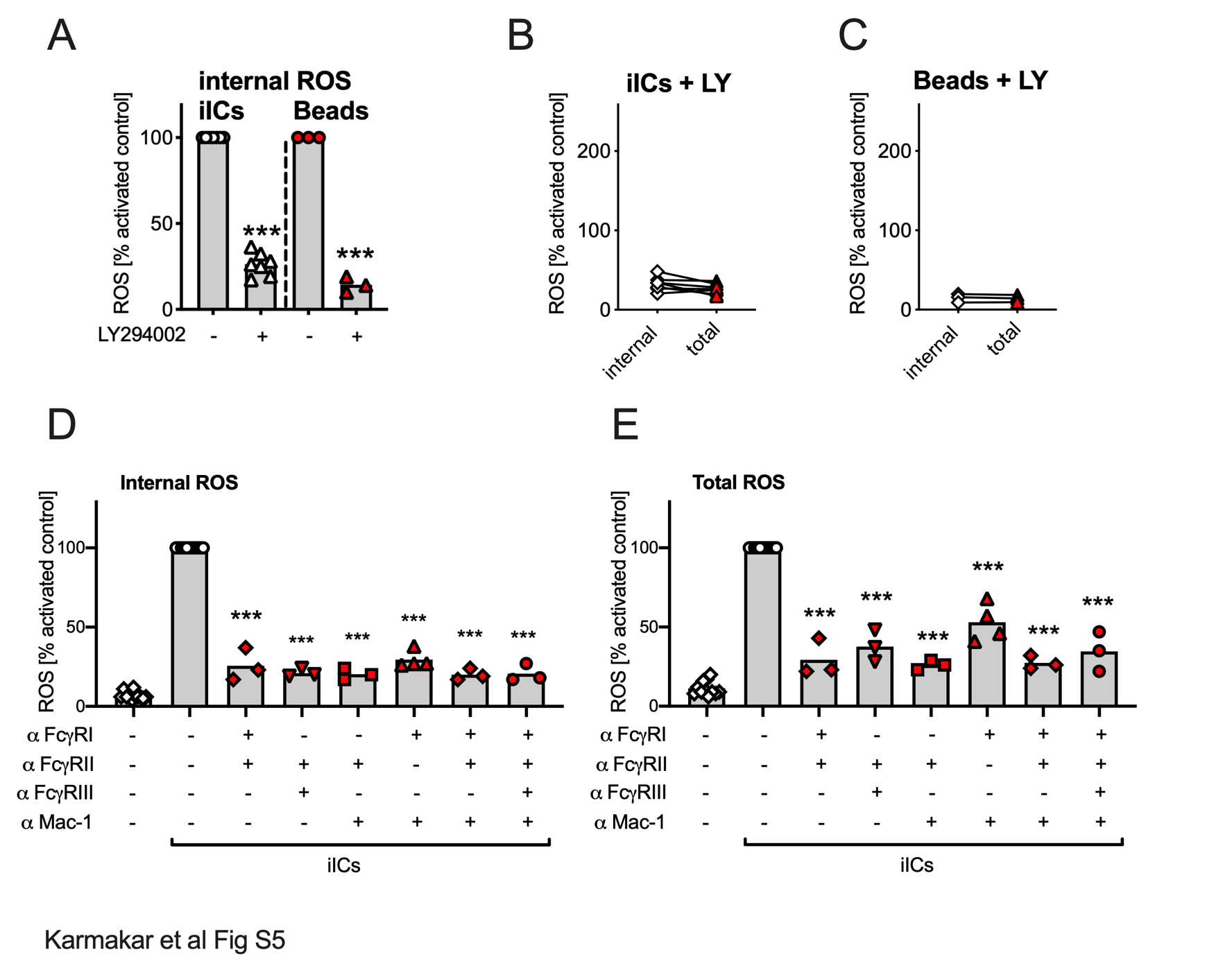

Supplement: Supplementary file 7 — Figure S5 [file 41419_2021_3528_MOESM7_ESM.png]

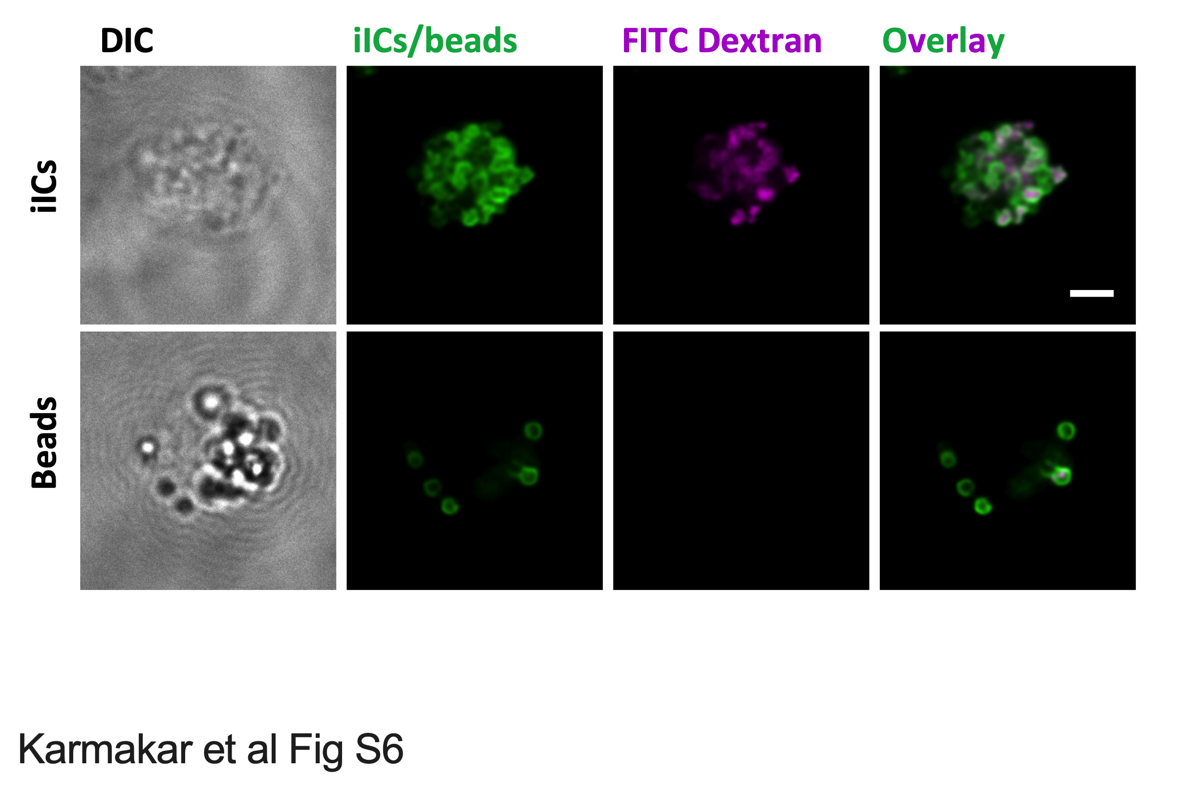

Supplement: Supplementary file 8 — Figure S6 [file 41419_2021_3528_MOESM8_ESM.png]

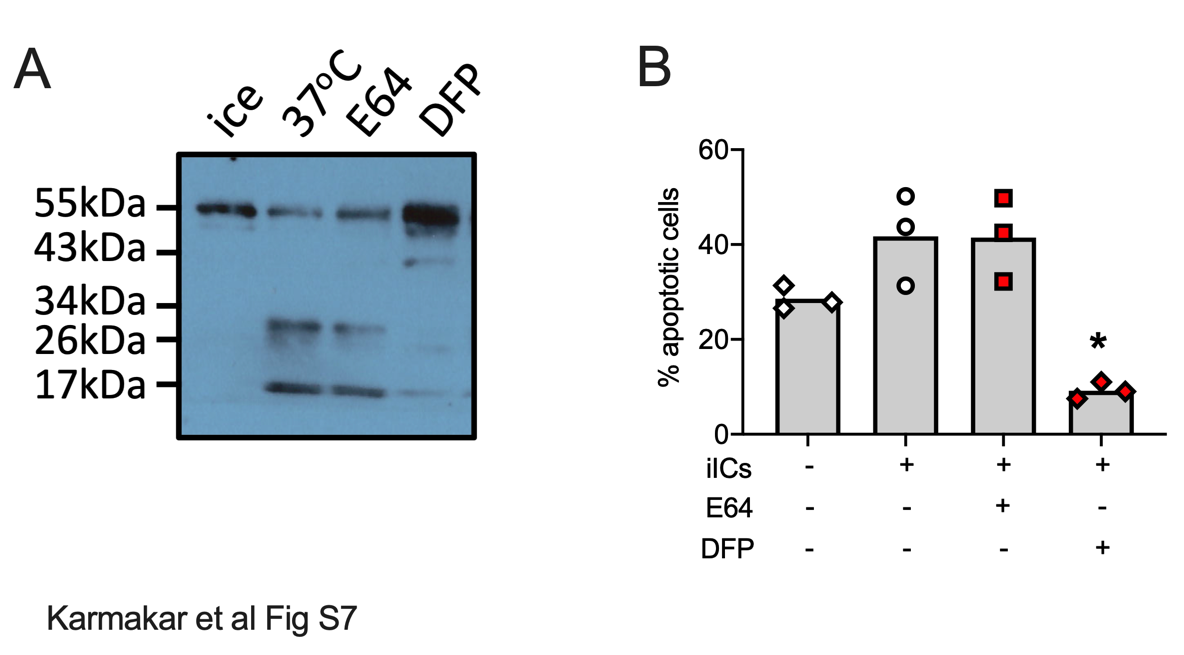

Supplement: Supplementary file 9 — Figure S7 [file 41419_2021_3528_MOESM9_ESM.png]
